# 

**Published:** 1880-04

**Authors:** 


Vol. 1.] TWO DOLLARS A YEAR. SINGLE COPIES 50 CENTS. [No. 2.

### THE ARCHIVES

OF

### COMPARATIVE MEDICINE AND SURGERY.

A Quarterly Journal

OF THE

ANATOMY, PATHOLOGY, AND THERAPEUTICS OF THE LOWER ANIMALS.

S.G.O. INDEXED

APRIL, 1880.

NEW YORK:

W. L. HYDE & CO., PUBLISHERS, NO. 22 UNION SQUARE.

1880.

